# Seroprevalence of polyomaviruses BK and JC in Finnish women and their spouses followed-up for three years

**DOI:** 10.1038/s41598-023-27850-7

**Published:** 2023-01-17

**Authors:** Hanna K. Laine, Tim Waterboer, Kari Syrjänen, Seija Grenman, Karolina Louvanto, Stina Syrjänen

**Affiliations:** 1 Department of Oral and Maxillofacial Diseases, Faculty of Medicine, University of Helsinki, P.O. Box 41, 00014 Helsinki, Finland; 2 Department of Oral Pathology and Radiology, Faculty of Medicine, University of Turku, Turku, Finland; 3 Division of Infections and Cancer Epidemiology, German Cancer Research Center (DKFZ), Heidelberg, Germany; 4 SMW Consultants Ltd, Kaarina, Finland; 5 Department of Obstetrics and Gynecology, University of Turku, Turku University Hospital, Turku, Finland; 6 Department of Obstetrics and Gynecology, Faculty of Medicine and Health Technology, Tampere University, Tampere, Finland; 7 Department of Obstetrics and Gynecology, Tampere University Hospital, Tampere, Finland; 8 Department of Pathology, University of Turku, Turku University Hospital, Turku, Finland

**Keywords:** Diseases, Medical research

## Abstract

BK (BKPyV) and JC (JCPyV) polyomavirus infections are commonly subclinical and known infrequently to cause serious clinical diseases. Longitudinal follow-up studies regarding JCPyV and BKPyV serological outcomes are scanty. We analyzed JCPyV and BKPyV IgG-antibodies in 327 pregnant women and their 132 spouses, enrolled in the longitudinal Finnish Family HPV cohort at Turku University Hospital, Finland. Blood samples taken at baseline, and at 12-, 24-, and 36-month follow-up visits were analyzed for capsid protein VP1-antibodies using multiplex serology. Seroprevalence was constant for both BKPyV and JCPyV across the follow-up, varying between 95–99% and 59–68%, respectively, in women and between 96–97% and 66–72%, respectively, in their spouses. Seroconversion to BKPyV and JCPyV was detected in 15% and 18% of the women and in 13% and 19% of the men, respectively. Waning of BKPyV and JCPyV antibodies was infrequent, present in only 5% of the women (both viruses) and in 1.5% of the male spouses (only BKPyV). The number of lifetime sexual partners (*p* = *0.038*) was lower among JCPyV seropositive men. To conclude, seropositivity to BKPyV and JCPyV is common among marital couples in Finland, with only slight differences between genders. In men, the sexual behavior might be associated with JCPyV seroprevalence.

## Introduction

Human polyomavirus (HPyV) family comprises 13 members of non-enveloped DNA viruses with circular double-stranded genome varying from 5100 to 5400 base pairs^1^. First two HPyVs were identified in the 1970’s; BK polyomavirus (BKPyV)^2^ and John Cunningham polyomavirus (JCPyV)^3^. BKPyV and JCPyV infections are generally subclinical but can cause also serious clinical diseases especially among immunocompromised individuals. BKPyV infection can lead to polyomavirus-associated nephropathy in renal transplant patients, while JCPyV has a causative role in progressive multifocal leukoencephalopathy (a fatal central nervous system demyelinating disease). Both BKPyV and JCPyV are capable of transforming cells in vitro^4–7^*.* In malignant transformation, both the viral large- and small T antigens play a key role. BKPyV and JCPyV are considered as possible human carcinogens (Group 2B) by IARC (https://monographs.iarc.who.int/list-of-classifications), although their causative role in human carcinogenesis still remains controversial.

BKPyV and JCPyV have been intensively studied, but the natural history of these infections is still incompletely understood. Furthermore, no longitudinal studies on spouses exist. Horizontal transmission via mucosal surfaces or virus-containing fluids has been suggested. BKPyV transmission is believed to take place primarily via upper respiratory and oral routes^8,9^. Both BKPyV and JCPyV can persist in the urinary tract allowing their asymptomatic urinary shedding even in healthy subjects^10–12^.

In healthy adult population, BKPyV seroprevalence is around 90% while the JCPyV is lower, around 50–70^10,13–16^. Seroepidemiological studies suggest that primary BKPyV infection might be acquired earlier in life than JCPyV, implicating a different mode of transmission^17,18^. Pregnancy has also shown to be associated with impaired immunocompetence, and viral replication and shedding of BKPyV increase during pregnancy^19–22^. However, there is only one previous study on pregnancy so far, with a single focus on BKPyV and JCPyV serology during the first and second pregnancy but not after pregnancy^14^.

The seroprevalence for BKPyV and JCPyV has varied widely among different studies, possibly explained by the different sensitivity of the assays used. Early studies until late 1990’s, were mostly based on the haemagglutination-inhibition (HI) method. The methods based on virus-like particle (VLP) ELISA became more common since the early 2000’^14,23^. Subsequently, multiplex detection with glutathione S-transferase (GST)-tagged proteins utilizing the Luminex platform become more popular^13,15,16,24,25^, allowing simultaneous analyses of multiple antibodies and more importantly also the comparison of the results provided by different studies.

As longitudinal studies are nearly lacking, the goal of our study was to determine the seroprevalence, seroconversion, and waning of antibodies to capsid protein VP1 of JCPyV and BKPyV among spouses during their 3-year prospective follow-up and to determine co-factors that could be associated with seropositivity.

## Materials and methods

### Participants

In total, 327 of the 329 women and 132 of their 133 male spouses enrolled originally in the Finnish Family HPV Study (FFHPV), were eligible for the present study. All women were pregnant at their 3^rd^ trimester when enrolled at the Maternity Unit of Turku University Hospital (Finland) as described earlier by Rintala et al. 2005^26^. All women filled in a standardized questionnaire providing comprehensive information on their demographics^26^. During their first post-partum visit 1.5–4.9 months after delivery, also the spouses of the enrolled mothers completed a standardized questionnaire. These comprehensive questionnaires collected demographic data e.g. on education, employment, marital status, number of sexual partners, alcohol consumption, smoking history and snuff usage, allergy, and atopy. Mean age of the mothers and their male spouses at enrollment was 25.5 years (range, 18–38 years) and 29.0 years (range 19–64 years), respectively. The study was approved by the Research Ethics Committee of Turku University Hospital (#3/1998, with amendments 45/1801/ 2018). All methods were carried out in accordance with relevant guidelines and regulations. Informed consent was obtained from all participants.

### Serology

#### Blood samples

Blood samples were taken at baseline and at 12-, 24-, and 36 months of follow-up as described earlier^27^. The number of blood samples of women and their male spouses available for the present study at 12-, 24- and 36-month visits were 278, 220, 261 and 112, 87, 94, respectively. After collection, the samples were centrifuged at 2400 r.p.m. for 10 min (Sorvall GLC-2; DuPont Instrument). Each serum sample was immediately divided into three 1 ml aliquots and stored first at −20 C for no longer than 1 week and then at −70 C until shipped on dry ice to DKFZ, Heidelberg, Germany, for serological analysis.

#### Serology analysis

The samples were analysed for IgG-antibodies to VP1 of BKPyV and JCPyV. Antonsson et al. and Gossai et al. have described the method for BKPyV and JCPyV serology used here in more detailed, earlier^15,16^. The method is a GST-capture ELISA^28^ combined with fluorescent bead technology^29^. In the present study, sera were scored as positive when the antigen-specific mean fluorescence intensity (MFI) values were greater than the cut-off level of 400 or 1000 MFI (stringent) for VP1 antigen of BKPyV and JCPyV.

#### Seroconversion

Seroconversion was defined by two conditions, both of which must be fulfilled: (1) at least a twofold increase of the previous serum MFI value and (2) MFI value exceeding over the cut-off of 400 MFI. Similarly, antibody waning was defined by two conditions: (1) at least a twofold decrease of the previous serum value, and (2) fall of the MFI value below the cut-off of 400 MFI.

### Statistical analysis

We first evaluated the seroprevalence, seroconversion, and waning of antibodies to capsid protein VP1 of JCPyV and BKPyV among the couples during their 3-year prospective follow-up using the criteria given above. All women were enrolled in the cohort while pregnant at 3^rd^ trimester. Thus, our study design allows us to study BKPyV and JCPyV seropositivity, antibody titers, seroconversion and antibody decay both during and after pregnancy. Demographic data recorded by the questionnaire at enrollment (women pregnant at 3rd trimester) was used to evaluate to the association between different co-factors and BKPyV/JCPyV seropositivity. The same data was analyzed also in male spouses.

All statistical analyses were run by using the SPSS for Windows (version 28.0.1.1; SPSS, Inc.) software package. Conventional 2 × 2 tables were used to analyze categorical variables, tested using the likelihood-ratio test or Fisher’s exact test. Differences in the means of continuous variables were analyzed by ANOVA or Mann–Whitney/Kruskal–Wallis test for two and multiple independent samples, respectively. All statistical tests performed were two-sided and declared significant when *p* < 0.05.

## Results

The mean and median levels of BKPyV and of JCPyV IgG-antibody levels of the pregnant women and their male spouses during their 3-year followed-up are summarized in Table 1. Both antibody levels showed a wide range. The mean and median antibody levels to BKPyV were higher than to JCPyV. Furthermore, we found a difference between genders in that the antibody levels of BKPyV were higher in women than in their spouses, while contrary was true for JCPyV antibody levels.

**Table 1:** Serum IgG-antibody levels of BK (BKPyV) and JC (JCPyV) polyomaviruses in pregnant women at their 3rd trimester and their male spouses from the Finnish Family HPV study at baseline and at the 12-, 24-, and 36- month follow-up visits. Antibody levels were given as median fluorescence intensity (MFI) values (Mean ± SD, Median and Range**)**.

	Baseline	12 mo	24 mo	36 mo
BKPyV MFI				
* Women (n)*	*327*	*278*	*220*	*261*
Mean ± SD	8497 ± 416	8993 ± 5126	8675 ± 4978	7732 ± 3854
Median	8640	9518	9276	7640
Range	72–20,773	51–20,198	26–20,276	136–19,141
* Male spouses (n)*	*132*	*112*	*87*	*94*
Mean ± SD	6965 ± 5053	8319 ± 5130	7908 ± 4910	7471 ± 4558
Median	6206	8132	7622	7158
Range	31–16,700	35–19,097	35–18,311	153–18,464
JCPyV (MFI)				
* Women (n)*	*327*	*278*	*220*	*261*
Mean ± SD	2340 ± 2901	2430 ± 2996	2391 ± 2885	2241 ± 2418
Median	765	870	759	1122
Range	0–15,007	0–13,921	0–10,191	4–12,117
* Male spouses (n)*	*132*	*112*	*87*	*94*
Mean ± SD	2573 ± 2939	3022 ± 3152	3101 ± 3359	3030 ± 2714
Median	1252	1901	2197	2813
Range	2–13,261	8–13,430	11–14,625	13–11,226

### Seroprevalence

**Figure 1:**
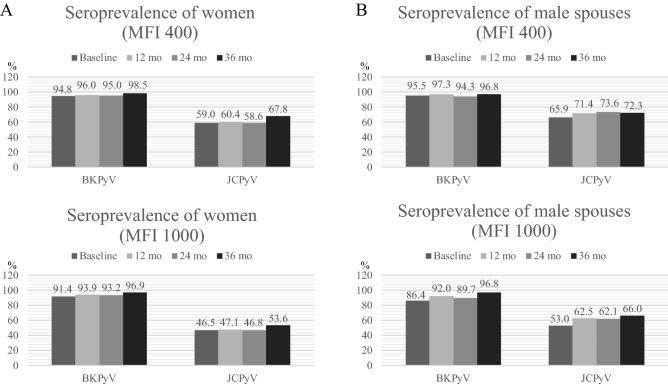
Seroprevalence (%) of BK (BKPyV) and JC (JCPyV) polyomaviruses among women (**A**) and their male spouses (**B**) at baseline (women at 3rd trimester pregnancy) and during the follow up at 12-, 24-, and 36-month visits. Upper panels show the seropositivity of BKPyV and JCPyV using MFI 400 as cut-off, while the lower panel shows the seroprevalences with the more stringent cut –off, MFI 1000.

Figure 1 shows seroprevalence (i.e., percentage of seropositive) of women and men at baseline and after one-, two-, and three years of follow-up. Seroprevalence of BKPyV was high in both genders and variation between different time points was limited, irrespective whether the 400 MFI or 1000 MFI cut-offs were used. The use of more stringent cut-off reduced the seroprevalence of BKPyV by 2–3% and 5–9% in women and their spouses, respectively. Seroprevalence of BKPyV was lowest among mothers to come at baseline (94.8%, MFI 400) and highest after three years of follow up (98.5%, MFI 400). Among the male spouses, the lowest and the highest seroprevalence of BKPyV was detected at the 2-year and 1-year visit; 94.3% and 97.3% (MFI 400), respectively.

Similarly as with BKPyV, the seroprevalence of JCPyV (MFI 400) was lowest in mothers to come at baseline visit (59.0%) and at the 2-year visit (58.6%). Nearly 70% of the women were seropositive for JCPyV at the last 3-year visit. Among the male spouses, seroprevalence of JCPyV (MFI 400) was lowest at the baseline (65.9%) and highest after two years (73.6%). JCPyV seropositivity was slightly less common in women than in their male spouses. As compared with BKPyV, seroprevalence of JCPyV was more sensitive to the cut-off change resulting in 14% and 6–12% decline in women and their spouses, respectively.

The association between demographic data and BKPyV and JCPyV seropositivity in women and their spouses are shown in Supplement Table 1. The only statistically significant association was found in men. The number of sexual partners when graded into four categories was significantly different (*p* = *0.038*) between JCPyV seroconverted and non-converted men. However, JCPyV seropositivity did not associate with the increase of lifetime sexual partners. Education, employment, smoking, alcohol consumption, using of snuff, marital status, allergy, and atopy did not show any association with BKPyV or JCPyV seropositivity.

### Seroconversion

**Figure 2:**
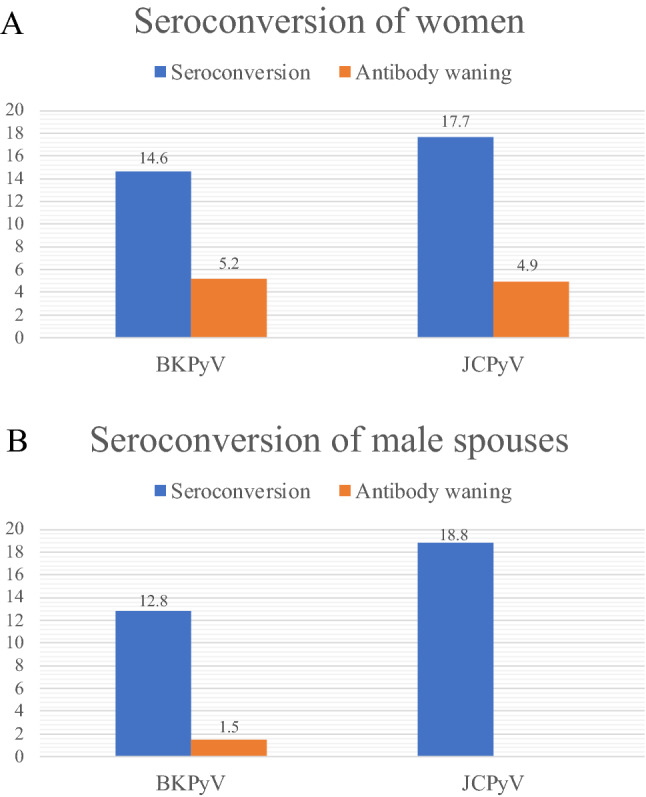
Proportion of the women (**A**) and their male spouses (**B**) who demonstrated BK (BKPyV) and JC (JCPyV) polyomavirus seroconversion or antibody waning during their 3-year follow-up. The number of women and their spouses who had BKPyV and JCPyV seroconversion were 48 and 58 and 17 and 25, respectively. The number of women and their spouses who had BKPyV and JCPyV antibody waning were 17 and 16 and 2 and 0, respectively.

The proportion of women and their male spouses who seroconverted for BKPyV or JCPyV during the follow up are presented in Fig. 2. Seroconversion for BKPyV was slightly more prevalent in women than in their spouses, while seroconversion for JCPyV was more prevalent in men. Waning of BKPyV and JCPyV antibodies was rare found only in 5% of the women. In their spouses, none had antibody waning for JCPyV, and BKPyV antibodies waned in 1.5% only during the follow-up.

The effect of pregnancy on BKPyV and/or JCPyV seropositivity could be assessed by comparing the seropositivity of women at their 3^rd^ trimester pregnancy with their seropositivity one year later (Table 2). In total, 56.7% of the pregnant women were seropositive for both BKPyV and JCPyV, while one year later the corresponding percentage was 58.8%. Of the pregnant women, 2.9% were seronegative for both viruses, while one mother became seropositive for both viruses in one year.

**Table 2:** The effect of pregnancy on BK (BKPyV) and/or JC (JCPyV) polyomavirus seropositivity assessed by comparing the seropositivity of 277 women at their 3rd trimester pregnancy (baseline) with their seropositivity one year later (12- month visit).

Seropositivity (MFI > 400)	Baseline n (%)	12-month visit N (%)
BKPyV + JCPyV +	157 (56.7)	163 (58.8)
BKPyV + JCPyV−	106 (38.2)	103 (37.2)
BKPyV− JCPyV +	6 (2.2)	4 (1.4)
BKPyV− JCPyV−	8 (2.9)	7 (2.5)
Total	277 (100.0)	277 (100.0)

## Discussion

To our knowledge, this is the first study evaluating BKPyV and JCPyV serology among spouses in a longitudinal setting. We showed that over 94% of the couples were seropositive for BKPyV, whereas JCPyV seroprevalence was lower, varying between 59–68% and 66–72% among women and their male spouses, respectively. Our results are in perfect alignment with those by Antonsson and coworkers who reported 96% seroprevalence for BKPyV and 60% and for JCPyV in an Australian population^15^. Importantly, they used the same Luminex platform with the same 400 MFI cut-off, as used in the present study. In addition, the antibody levels reported as MFI-values were similar to those in our cohort. Gossai and coworkers made a study on BKPyV, JCPyV and other polyomaviruses in a US population also using the same Luminex platform but with a lower cut-off (MFI 250) and higher sample dilution (1:1000)^16^. They found no gender differences in BKPyV seropositivity (88%), while seroprevalence of JCPyV was lower in women (49%) than in men (60%), exactly as shown in our study. Several studies have confirmed that seropositivity for JCPyV increases with age especially among men^11,16,30^. In the present cohort, these couples were healthy adults with the mean age of 25 years (women) and 29 years (men), thus precluding calculations of age-specific data. In general, JCPyV seroprevalence was higher in the present study than reported by earlier studies^11,16,17^. The JCPyV seroprevalence found here is similar as reported earlier in age groups older than 50 years^11,16^. However, Viscidi et al. reported findings closely matching with our data, the seroprevalence of JCPyV being 60–68% in the age group of 20–49 years^31^.

Stolt and coworkers described that BKPyV seropositivity increased rapidly with increasing age of the children, reaching 99% seroprevalence at 7–9 years of age. This was followed by approximately 10–20% decline, attributed to the antigenic variants of BKPyV. In contrast, seroprevalence of JCPyV increased more slowly with increasing age being highest among the mothers over 25 years of age (72%)^14^. As expected, this seroprevalence was very close to that in our cohort, because the study subjects in this study represented also mothers to be. Similarly as reported by Antonsson et al.^15^, we also found that overall BKPyV seropositivities were relative robust to changes in the MFI cut-offs used, i.e., 1000 MFI or 400 MFI. JCPyV seroprevalence was more sensitive to cut-off changes than BKPyV as described also by Antonsson et al.^15^. Interestingly, seroprevalence for JCPyV declined more in women than in men. We could also confirm that among the seropositive individuals, there was a marked fluctuation in the MFI values as also reported by Antonsson et al.^15^.

In our study, we also found seroconversion for BKPyV and JCPyV in both women and their male spouses during their 3-year follow-up. Seroconversion for BKPyV was of similar magnitude in both genders (13% in men and 15% in women). It was shown also for JCPyV, albeit seroconversion of JCPyV was slightly more prevalent (19% men and 18% women) than that of BKPyV. We also detected BKPyV antibody waning in 5% of these couples while JCPyV antibody waning was found only among women (2%). Stolt and coworkers have studied seroconversion or antibody waning of BKPyV and JCPyV among Finnish pregnant women previously. They did not find any seroconversion or loss of seropositivity during 5-year follow up^14^. This discrepancy can be explained partly by the use of different assays for BKPyV and JCPyV serology. We used GST-capture ELISA in combination with fluorescent bead technology, while Stolt et al. used VLP-based ELISA^14^. Another source of discordant data can be due to the different criteria used to define seroconversion. Importantly, we did not look only seroconversion from seronegative to seropositive exceeding the 400 MFI cut-off but seroconversion was recorded when there was at least a twofold increase of the previous serum MFI value (e.g. from 1000 to 2000MFI or 4000 MFI to 8000MFI). Antonsson and co-workers found in their longitudinal study that none of the subjects seroconverted to BKPyV while serorevision was found in 1% and fluctuation in 2% of the participants. According to their data, seroconversion for JCPyV was more prevalent and found in 4%. They reported that seroconversion and fluctuation were more prevalent, detected in 4% and 3%, respectively^15^. Our study confirmed their findings implicating that JCPyV antibody status over time was less stable than that of BKPyV.

All previous studies support the view that the exposure to BKPyV occurs in early life because seroprevalence studies indicate that nearly 50% of children at the age of 2 years are seropositive, and more than 90% of children have acquired seropositivity by the age of 10 years. This pattern of seropositivity support the view that BKPyV transmission primarily takes place by upper respiratory and oral routes^8,9^. On the contrary, JCPyV seroprevalence increases slowly and markedly later than for BKPyV, reaching 50–80% among healthy adults. The previous evidence supports the view that exposure to JCPyV occurs mostly in adult life.

This different age profile between JCPyV and BKPyV exposure might indicate that different risk factors are being involved in developing seropositivity for these two viruses. The only covariates recognized so far are age and gender, the latter being still controversial^15,16^. Only few studies have analyzed other risk factors associated with JCPyV seropositivity or antibody stability^15,16^. As far as the authors are aware, the present study seems to be the first to find evidence that sexual behavior, determined by the number of sexual partners when graded into four different categories, was different between JCPyV seroconverted and non-converted men. JCPyV is known to be asymptomatically and intermittently shedding in the urinary tract even with high viral load^11,32^.However in the current study, the number of lifetime sexual partner was lower among JCPyV seropositive men. In the present analysis, education, employment, smoking, alcohol consumption, using of snuff, allergy, atopy, or glucocorticoid use did not have any significant association in predicting seropositivity either for BKPyV or JCPyV. In line with our results, two previous studies have confirmed that smoking had no association with BKPyV or JCPyV serostability or seropositivity^15,16^.

We also demonstrated that the index pregnancy (FFHPV cohort) had no impact on the profile of either the BKPyV or JCPyV serology among the mothers. One would expect a rise in BKPyV seropositivity as BKPyV reactivation has been documented in up to 53% of pregnant women through PCR amplification of viral sequences^19–22^. We found that the mean antibody levels of BKPyV were lower in pregnant mothers at their 3rd trimester (and their spouses as well) as comparted with the antibody levels one year after the pregnancy. One could speculate that reactivation or reinfection of BKPyV among spouses during the pregnancy could result in elevated antibody levels. Stolt and coworkers also studied BKPyV and JCPyV serology in pregnant mothers (at their 1^st^ trimester) and later when they were pregnant for the second time. However, they could not estimate the effect of pregnancy because all samples were derived from pregnant women^14^. In addition, McClure et al. have showed that asymptomatic urinary shedding of BKPyV was more common in pregnant women than in non-pregnant, although there was no difference in the level of JCPyV excretion^33^.

Taken together, the present longitudinal cohort study demonstrates that both BKPyV and JCPyV are ubiquitous among young healthy couples, with only minor gender-related differences.

## Supplementary Information

Supplementary Information.

## Data Availability

All data generated and analyzed during current study are included in this article.
