# Supplementary material for: Seroprevalence of polyomaviruses BK and JC in Finnish women and their spouses followed-up for three years
Source: Sci Rep. 2023 Jan 17;13:879. doi: 10.1038/s41598-023-27850-7 (PMC9845201; doi:10.1038/s41598-023-27850-7)
Supplement: Supplementary file 1 — Supplementary Information. [file 41598_2023_27850_MOESM1_ESM.pdf]

Supplement table 1. Questionnaire-recorded demographic data associated with BK (BKPyV) and JC (JCPyV) polyomavirus seropositivity in women and their male spouses.

Seropositivity	Women' responses BKPyV/JCPyV	BKPyV Yes	BKPyV No	P-value	JCPyV Yes	JCPyV No	p-value
**Education**	270/271			0.719			0.076
Comprehensive school		4(9%)	21(9%)		3(6%)	22(10%)	
Technical school		11(23%)	64(29%)		22(41%)	51(24%)	
Undergraduate		10(21%)	35(16%)		5(9%)	42(19%)	
Institute		14(30%)	76(34%)		16(30%)	71(33%)	
University		8(17%)	27(12%)		8(15%)	31(14%)	
**Employment**	265/265			0.840			0.597
Employed		27(59%)	138(63%)		35(65%)	131(62%)	
Unemployed		11(24%)	49(22%)		13(24%)	45(21%)	
Student		8(17%)	32(15%)		6(11%)	35(17%)	
**Marital status**	270/271			0.717			0.771
Married		23(49%)	102(46%)		26(48%)	98(45%)	
Not married		3(6%)	15(7%)		5(9%)	14(7%)	
Common-law marriage		20(43%)	104(47%)		23(43%	102(47%)	
Divorced		1(2%)	2(1%)		0(0%)	3(1.5%)	
**Number of sexual partners**	269/270			0.386			0.290
0-2		7(15%)	56(25%)		14(26%)	54(25%)	
3-5		15(32%)	74(33%)		21(40%)	66(30%)	
6-10		13(28%)	49(22%)		7(13%)	54(25%)	
>10		12(26%)	43(19%)		11(21%)	43(20%)	
**Smoking**	269/270			0.517			0.290
Non-smoker		23(49%)	110(50%)		26(48%)	107(50%)	
1-10 cigarettes a day		12(26%)	65(29%)		13(24%)	66(31%)	
11-20		10(21%)	44(20%)		15(28%)	38(18%)	
>20		2(4%)	3(1%)		0(0%)	5(2%)	
**Alcohol**	269/270			0.272			0.753
Never		5(11%)	22(10%)		4(7.5%)	23(11%)	
1 dose a day		0(0%)	1(0.5%)		0(0%)	1(0.5%)	
1 dose 2-3 times a week		1(2%)	27(12%)		4(8%)	22(10%)	
1 dose a week		15(32%)	66(30%)		20(38%)	64(30%)	
1 dose a month		26(55%)	106(48%)		25(47%)	107(49%)	
**Snuff**	244/245			1.000			1.000
Non-user		43(100%)	200(99.5%)		50(100%)	194(99.5%)	
Pack per month		0(0%)	1(0.5%)		0(0%)	1(0.5%)	
**Allergy**	267/268			0.159			0.226
No		21(46%)	126(57%)		33(62%)	114(53%)	
Yes		25(54%)	95(43%)		20(38%)	101(47%)	
**Atopy**	260/261			0.259			0.085
No		35(78%)	182(85%)		47(92%)	173(82%)	
Yes		10(22%)	33(15%)		4(8%)	37(18%)	

Seropositivity	Male Spouses´ responses BKPyV/JCPyV	BKPyV Yes	BKPyV No	P-value	JCPyV Yes	JCPyV No	p-value
**Education**	122/124			0.395			0.472
Comprehensive school		0(0%)	9(9%)		2(8%)	7(7%)	
Technical school		7(44%)	43(41%)		7(28%)	44(44%)	
Undergraduate		0(0%)	12(11%)		4(16%)	9(9%)	
Institute		7(44%)	27(26%)		9(36%)	25(25%)	
University		2(13%)	15(14%)		3(12%)	14(14%)	
**Employment**	121/123			0.451			0.444
Employed		15(88%)	89(86%)		23(92%)	82(84%)	
Unemployed		0(0%)	7(7%)		0(0%)	8(8%)	
Student		2(12%)	8(8%)		2(8%)	8(8%)	
**Marital status**	123/125			0.484			0.084
Married		12(71%)	56(53%)		17(68%)	52(52%)	
Not married		0(0%)	1(1%)		1(4%)	0(0%)	
Common-law marriage		5(29%)	48(45%)		7(28%)	47(47%)	
Divorced		0(0%)	1(1%)		0(0%)	1(1%)	
**Number of sexual partners**	122/124			0.618			**0.038**
0-2		1(6%)	14(13%)		5(20%)	11(11%)	
3-5		2(13%)	25(24%)		3(12%)	24(24%)	
6-10		4(25%)	24(23%)		10(40%)	18(18%)	
>10		9(56%)	43(41%)		7(28%)	46(47%)	
**Smoking**	120/122			0.741			0.574
Non-smoker		10(63%)	65(63%)		15(60%)	62(64%)	
1-10 cigarettes/day		1(6%)	15(14%)		2(8%)	14(14%)	
11-20		3(19%)	16(15%)		6(24%)	13(13%)	
>20		2(13%)	8(8%)		2(8%)	8(8%)	
**Alcohol**	121/123			0.068			0.880
Never		0(0%)	2(2%)		0(0%)	3(3%)	
1 dose a day		1(6%)	3(3%)		1(4%)	3(3%)	
1 dose 2-3/ week		10(63%)	31(30%)		10(40%)	32(33%)	
1 dose a week		4(25%)	43(41%)		8(32%)	39(40%)	
1 dose a month		1(6%)	26(25%)		6(24%)	21(21%)	
**Snuff**	113/115			1.000			1.000
Non-user		0(0%)	1(1%)		25(100%)	83(92%)	
Pack per month		13(100%)	93(93%)		0 (0%)	3 (3%)	
2 packs per month		0(0%)	3(3%)		0(%)	2(3%)	
**Allergy**	119/121			0.702			0.820
No		11(65%)	61(60%)		14(56%)	58(60%)	
Yes		6(35%)	41(40%)		11(44%)	38(40%)	
**Atopy**	118/120			0.324			0.361
No		15(88%)	95(94%)		22(88%)	90(95%)	
Yes		2(12%)	6(6%)		3(12%)	5(5%)	
